# Cold Atmospheric Plasma Modified Electrospun Scaffolds with Embedded Microspheres for Improved Cartilage Regeneration

**DOI:** 10.1371/journal.pone.0134729

**Published:** 2015-07-29

**Authors:** Wei Zhu, Nathan J. Castro, Xiaoqian Cheng, Michael Keidar, Lijie Grace Zhang

**Affiliations:** 1 Department of Mechanical and Aerospace Engineering, The George Washington University, Washington, District of Columbia, United States of America; 2 Department of Medicine, The George Washington University, Washington, District of Columbia, United States of America; University of Wisconsin-Madison, UNITED STATES

## Abstract

Articular cartilage is prone to degeneration and possesses extremely poor self-healing capacity due to inherent low cell density and the absence of a vasculature network. Tissue engineered cartilage scaffolds show promise for cartilage repair. However, there still remains a lack of ideal biomimetic tissue scaffolds which effectively stimulate cartilage regeneration with appropriate functional properties. Therefore, the objective of this study is to develop a novel biomimetic and bioactive electrospun cartilage substitute by integrating cold atmospheric plasma (CAP) treatment with sustained growth factor delivery microspheres. Specifically, CAP was applied to a poly(ε-caprolactone) electrospun scaffold with homogeneously distributed bioactive factors (transforming growth factor-β1 and bovine serum albumin) loaded poly(lactic-co-glycolic) acid microspheres. We have shown that CAP treatment renders electrospun scaffolds more hydrophilic thus facilitating vitronectin adsorption. More importantly, our results demonstrate, for the first time, CAP and microspheres can synergistically enhance stem cell growth as well as improve chondrogenic differentiation of human marrow-derived mesenchymal stem cells (such as increased glycosaminoglycan, type II collagen, and total collagen production). Furthermore, CAP can substantially enhance 3D cell infiltration (over two-fold increase in infiltration depth after 1 day of culture) in the scaffolds. By integrating CAP, sustained bioactive factor loaded microspheres, and electrospinning, we have fabricated a promising bioactive scaffold for cartilage regeneration.

## Introduction

Articular cartilage is a connective tissue found in diarthrodial joints which provides a lubricated surface between bones, allowing load transfer while withstanding repetitive loading over a person’s lifetime. It is a thin layer of tissue on the order of 1–4 mm in thickness depending on its location within the human body, thus rendering it prone to degeneration as a result of trauma, disease, and mechanical overloading [1]. Injuries to cartilaginous tissue can be classified into three categories: superficial matrix disruption, partial, and full thickness defects. Amongst them, superficial matrix disruption as a result from blunt trauma can be repaired by human chondrocytes, but partial thickness defects and full thickness defects are inherently incapable of self-repair. Therefore, artificial intervention is required to repair injured cartilage under these conditions. Cartilage regeneration primarily occurs when progenitor cells or chondrocytes are accessible and undergo chondrogenesis to form tissue *de novo* [2]. Following this logic, multiple treatment options including microfracture, mosaicplasty, and autologous chondrocyte implantation have been explored [3]. However, there still remains an ongoing need for the development of novel strategies due to the poor long-term outcome of these traditional treatment methods. Tissue engineering as an alternative strategy has presented promise in repairing cartilage tissue defects [4].

3D tissue engineered scaffolds provide a template and environmental cues to support cell growth, proliferation, differentiation, and facilitate formation of new tissue. In particular, electrospinning is a versatile scaffold fabrication technique which has gained increased attention in the realm of cartilage tissue repair [3, 5]. This technique allows for the fabrication of fibrous scaffolds with biomimetic micro-to-nanoscale diameters closely resembling the porous architecture of native extracellular matrix. The versatility of electrospinning makes it possible to obtain scaffolds with controllable geometry, surface chemistry, and mechanical properties by adjusting the process parameters including polymer molecular weight and concentration, applied voltage, and flow rate.

A wide variety of biomaterials have been utilized in electrospinning to include poly(ε-caprolactone) (PCL) which is one of the most widely used synthetic polymers due to its excellent biocompatibility, biodegradability, and mechanical properties [6]. Studies have shown the capacity of PCL fibrous scaffolds to maintain chondrocyte phenotype and upregulate collagen type IIB splice variant transcript expression (indication of mature chondrocyte phenotype) [7]. Although PCL exhibits desirable characteristics, the materials’ inherent hydrophobic nature leads to the absence of surface cell-recognition sites limiting its biomedical application [8]. In an effort to improve cell-scaffold interactions, approaches such as the incorporation of bioactive peptides or proteins, alkaline hydrolysis, blending with other biopolymers, and processing with plasma deposition have been carried out [9–13]. However, most of these surface modification techniques are costly, time consuming, and routinely impair the bulk bioactive properties of the scaffold. Therefore, a more effective surface modification technique which can maintain the scaffolds’ bioactive properties while improving cell growth is still desirable.

In an effort to develop such a novel scaffold surface modification technique, we introduce cold atmospheric plasma (CAP) in this study targeting to render electrospun scaffold with more bioactivity and cell-favorable surface properties. Unlike traditional high temperature plasmas, CAP is an ionized gas whose temperature is close to room temperature composed of a unique environment of charged particles, including reactive oxygen and nitrogen species, photons, and electrostatic and electromagnetic fields [14]. These unique chemical and physical properties have enabled its recent application to biomedicine, including sterilization, biomaterial preparation, cancer therapy, and dental surgery [15–20]. CAP treatment can serve as an innovative and effective method to modify materials for particular biological applications. For instance, when utilizing CAP as a source of treating biological materials, it was demonstrated that CAP can specifically interact with organic materials without causing thermal/electric damage [21]. In our recent study, after CAP treatment, a lyophilized nanocrystalline hydroxyapatite/chitosan scaffold was induced with a more biomimetic and bioactive surface [22]. Greatly enhanced human marrow-derived mesenchymal stem cells (MSC) adhesion and osteogenic differentiation were observed in CAP modified scaffolds associated with increased total protein, collagen synthesis, and calcium deposition after 3 weeks of culture. Thus, it is postulated here that CAP modification can create a bioactive implantable cartilage scaffold for enhanced cell growth and tissue regeneration.

In addition to cell favorable surface chemistry, the incorporation of cell-specific bioactive factors within scaffolds is significant to improve cell growth and regulate cell behavior. Numerous studies have shown that bioactive factors incorporated within a scaffold matrix can control cellular function as well as promote successful tissue formation [23]. With this in mind, we fabricated poly(lactic-co-glycolic) acid (PLGA) microspheres with controlled bioactive factor delivery (bovine serum albumin (BSA) and transforming growth factor-β1 (TGF-ß1)) via a water/oil/water (w/o/w) double emulsion solvent extraction method for the enhancement of cell proliferation and induction of chondrogenesis. As a result, the biomimetic cartilage scaffold fabricated in this study integrates CAP, a sustained bioactive factor delivery system, and electrospinning for synergistic regulation of MSC growth, infiltration, and chondrogenic differentiation. Scaffold morphology, hydrophilicity, surface chemistry, protein adsorption, and mechanical properties were evaluated, and MSC growth and the induction of chondrogenesis were subsequently investigated.

## Materials and Methods

### Fabrication of Electrospun PCL Scaffold with PLGA Microspheres

**Fig 1:**
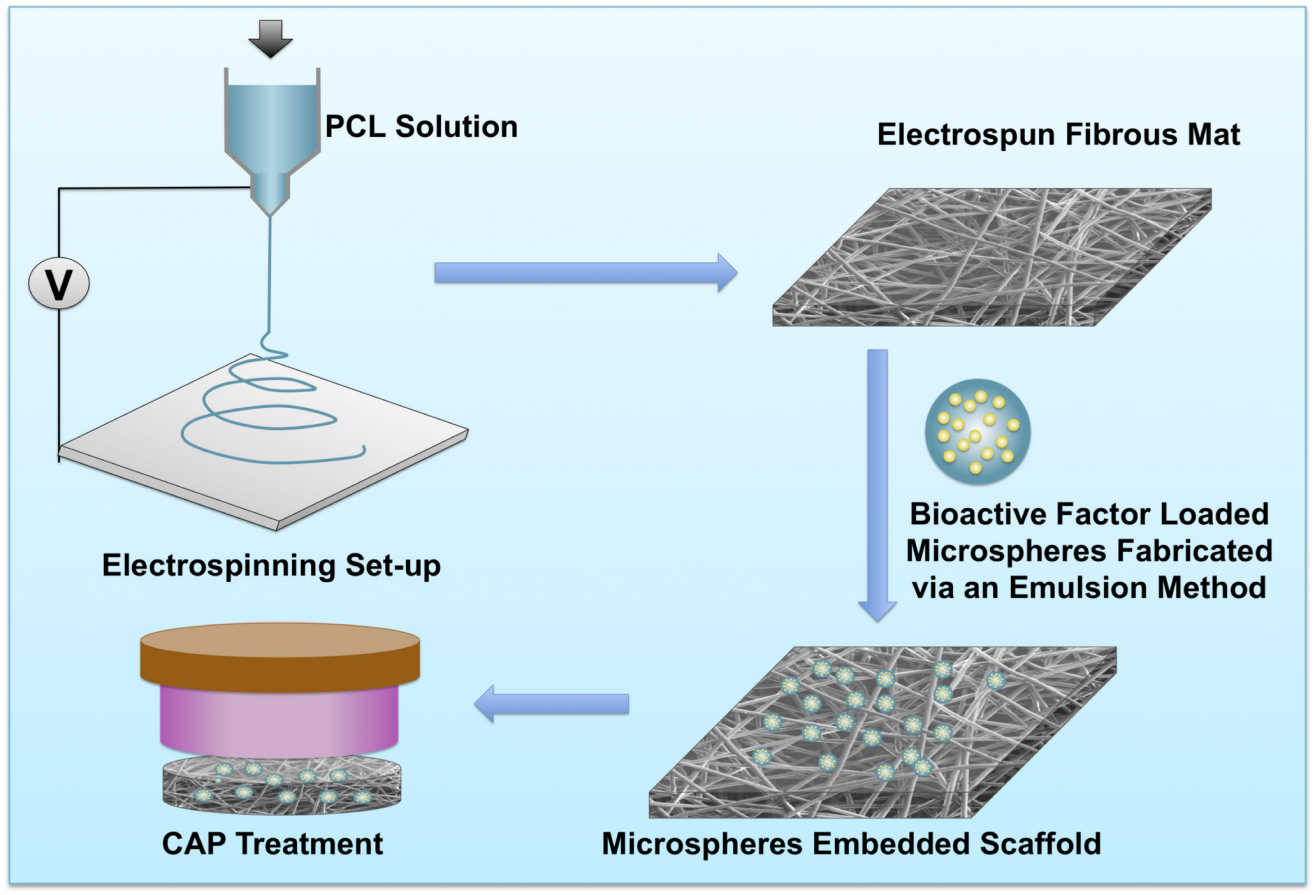
Schematic illustration of microspheres embedded electrospun cartilage scaffolds preparation and CAP modification.

Fig 1 illustrates the preparation of microsphere-embedded scaffolds with CAP surface modification. The electrospun polymer solution was prepared by dissolving PCL (Mw: 70,000–90,000) in chloroform at a concentration of 20% (w/v). The polymeric solution was then fed into a 10 mL standard syringe attached to a 26 G blunted stainless steel needle using a syringe pump at a flow rate of 4.5 mL.h^-1^ under an applied voltage of 7 kV. Electrospun fibers were collected on a metal plate wrapped with aluminum foil at a distance of 12 cm from the needle tip.

Bioactive factor encapsulated microspheres were synthesized by a modified w/o/w double emulsion solvent extraction method. Briefly, a BSA (10 mg.mL^-1^) or TGF-β1/BSA (250 ng.mL^-1^ TGF-β1 in 10 mg.mL^-1^ BSA) was dissolved in ultra pure water as the internal water phase. Oil phase comprised of 50 mg PLGA (50:50 with inherent viscosity range from 0.55 to 0.75 dL.g^-1^ in Hexafluoroisopropanol) in 1000 μL acetone. Then 500 μL BSA or TGF-β1/BSA aqueous solution was added into the PLGA solution and emulsified for 55 s using a sonicator at an output power of 500 W at 20 kHz. This first emulsion (w/o) was decanted into 10 mL ultra pure water to produce a double w/o/w emulsion. The microsphere containing solution was stirred for 30 min at room temperature to remove the organic solvent. For the introduction of microspheres, an electrospun fibrous mat was punched into 8 mm diameter test samples and placed in a 48-well plate. 500 μL of the microsphere suspension was injected to the well plate followed by shaking for 2 h.

### CAP Device

The CAP device employed in this study utilized the dielectric barrier discharge of helium gas which has been characterized in detail in our previous work.[18, 22] The electrodes for plasma production were composed of a 1 mm diameter central electrode wrapped with a grounded outer electrode of 4.5 mm diameter quartz tube. A high output voltage of 3.6 kV with a frequency of 13 kHz was applied to generate homogenous plasma through delivery of the helium gas. The flow rate of helium was kept constant at 5 L.min^-1^.

Scaffolds were treated by directly placing the scaffolds beneath the plasma electrode at a distance of 2 cm. Samples were exposed to CAP for 1 min, 3 min, and 5 min, respectively and characterized and evaluated for MSC culture studies.

### Characterization of Microspheres and Scaffolds

Microsphere morphology was examined by scanning electron microscopy (SEM, Zeiss NVision 40 FIB) at an accelerating voltage of 2 kV after sputter coating with gold. The size distribution of microspheres was determined from SEM micrographs using image analysis software (Image J, National Institutes of Health, USA). BSA encapsulation efficacy was tested by dissolving microspheres in 0.1 M NaOH aqueous and quantified using a protein assay reagent kit (Pierce Biotechnology).

The surface morphology of scaffolds was examined using SEM at 3 kV accelerating voltage with gold coating as well. A video contact angle system (DSA4, Krüss) was employed to determine the wettability (or hydrophilicity) of the electrospun scaffolds with and without CAP treatment. An ultra-pure water droplet of 0.9 μL was automatically deposited on the samples’ surface using a syringe. Three samples were measured for each test. Protein adsorption including fibronectin and vitronectin upon treated and untreated scaffolds was conducted by an enzyme-linked immunosorbent assay (ELISA). Briefly, scaffolds were incubated in Dulbecco’s modified Eagle’s medium (DMEM, Lonza) with and without fetal bovine serum (10%) (Atlanta Biologicals) for 24 h at 37°C. After washing with phosphate buffer saline (PBS) and blocking with 2% BSA for 1 h, the first antibody solution (fibronectin antibody, 1:100 or vitronectin antibody, 1:100, Thermo Scientific) was added to each scaffold. Goat anti-mouse lgG (H + L) (1:100 in 1% BSA, Life Technologies) serving as the secondary antibody was added prior to incubating for 1 h using Tween 20 (0.05% in PBS). Then, samples were incubated with 2,2’-azino-bis(3-ethylbenzothiazoline-6-sulphonic acid) (Vector Labs) solution for 20 min at room temperature in the dark followed by reading the absorbance at 405 nm by a spectrophotometer (Thermo Scientific Multiskan GO).

Compression testing of all samples (circular samples of 5 mm in diameter) was analyzed using an ATS uniaxial mechanical tester equipped with a 100 N load cell at a cross-head speed of 0.2 mm.s^-1^ under ambient condition.

### MSC Culture and *in vitro* Proliferation Study

Primary MSCs were harvested from healthy consenting donor’s iliac crest (Female; Age: 27) at Tulane University under an IRB-approved protocol with informed consent. And then they are distributed from the Texas A&M Health Science Center, Institute for Regenerative Medicine and are thoroughly characterized. We had the fully executed Material Transfer Agreement that was needed to obtain the cells, which provides verification that we in fact did not obtain the cells from the donors themselves. Passage number 3 to 6 was used for all of the following cell studies. MSCs were cultured in Alpha Minimum Essential medium (α-MEM, Gibco) supplemented with 16.5% fetal bovine serum, 1% (v/v) L-glutamine (Invitrogen), and 1% penicillin/streptomycin (Invitrogen) in a 75 cm^2^ cell culture flask. Cells were incubated at 37°C in a humidified atmosphere containing 5% CO_2_ and media was changed every other day.

For cell proliferation studies, circular specimens (8 mm in diameter) were sterilized under UV for 20 min, rinsed three times with PBS and subsequently prewet in media overnight prior to cell seeding. To test the efficiency of loaded bioactive factor (BSA), three sample groups (bare PCL, PCL with directly sprayed BSA, and PCL with BSA encapsulated microspheres) were prepared. In addition, samples with embedded microspheres were treated under varying CAP exposure times (0 min as control, 1 min, 3 min) and studied to evaluate the influence of CAP treatment on cell behavior. Cells were seeded at a density of 10,000 per scaffold. At predetermined time points, the cell number was quantified by a non-radioactive cell proliferation assay (MTS assay, Promega). The absorbance of each well was analyzed at 490 nm using a Thermo Scientific Multiskan GO spectrophotometer.

### Immunofluorescence Microscopy

Immunofluorescence was used to analyze MSC spreading and infiltration in the scaffolds after 1, 3, and 5 days culture. Samples were fixed with 10% formaldehyde for 10 min before rinsing 3× with PBS and permeabilizing with 0.05% Triton X-100 and double staining with Rhodamine-Phalloidin (Life Technologies) and 4’-6-diamidino-2-phenylindole (DAPI, Sigma-Aldrich) for F-actin and cell nucleus. Fluorescent images were visualized using a laser scanning confocal microscope (LSCM 710, Zeiss). Z-stack images were obtained to qualitatively evaluate cell migration throughout the scaffolds with infiltration depth quantitatively measured by ImageJ.

### MSC Chondrogenic Differentiation Study *In Vitro*

MSCs were seeded at a density of 100,000 cells/cm^2^ on each scaffold for chondrogenic differentiation studies. Seeded scaffolds were maintained in chondrogenic media composed of complete MSC media supplemented with 100 nM dexamethasone, 1% ITS+, 100 μg.mL^-1^ sodium pyruvate, 40 μg.mL^-1^ proline and 50 μg.mL^-1^ L-ascorbic acid 2-phosphate. After 1, 2, and 3 weeks of culture, samples were washed in PBS, freeze-dried, and enzymatically digested in Papain at 60°C for 18 h. Cartilage development was quantified on the basis of glycosaminoglycan (GAG) content, type II collagen, and total collagen synthesis.

GAG levels in digested solution were determined by binding of the acidic polymer to 1, 9-dimethylmethylene blue (DMB, Sigma-Aldrich). DMB solution was prepared by dissolving 16 mg DMB in 1 L water containing 3.04 g glycine, 1.6 g NaCl and 95 mL of 0.1 M acetic acid. 20 μL of digested samples was placed in a 96-well plate followed by the addition of 200 μL DMB solution. Samples were then shaken for 5 seconds and the absorbance was immediately measured at 524 nm.

Human type II collagen was evaluated via a type II collagen ELISA (TSZ ELISA) per the manufacturer’s instructions. Briefly, aliquots of digested sample were added to a pre-coated 96-well plate containing purified human type II collagen antibody. After 40 min of incubation at 37°C, samples were removed and rinsed with washing buffer. Biotinylated antibody was then added to each well and incubated for an additional 20 min. Enzyme was added followed by rinsing of the well plate with washing butter. Afterwards, a stop solution was sequentially added and the absorbance was read at 450 nm.

Total collagen synthesis was evaluated using a sirius red colorimetric assay. Specifically, sample aliquots were placed in a 96-well plate and dried overnight. Afterwards the samples were washed with distilled water then 150 μL of 0.1% Sirius red stain (Direct Red 80, Sigma) in saturated picric acid was added to each well and incubated for 1 hour at room temperature. Wells were washed four times with acidified water (5% acetic acid) and incubated with 150 μL 0.1 M NaOH for 30 minutes at room temperature. The solutions of each well were transferred to a new 96-well plate and read on a spectrophotometer at 550 nm.

### Statistical analysis

All quantitative experiments were run in triplicate and data are presented as mean ± standard error of the mean, unless indicated otherwise. Statistical analysis was carried out using student’s t-test. A value of p<0.1 was considered statistically significant.

## Results

### Preparation and Characterization of CAP Treated Scaffolds with Microspheres

**Fig 2:**
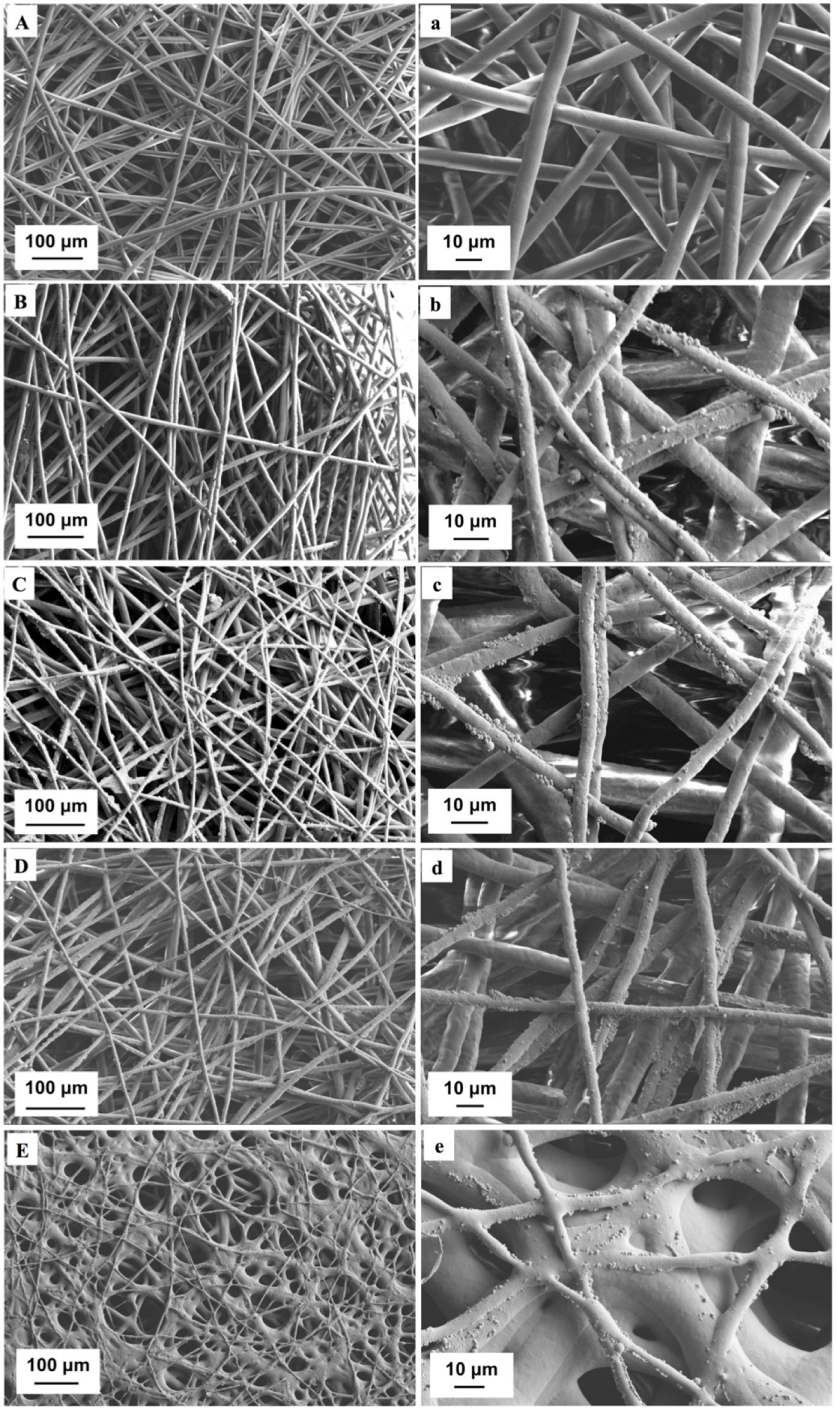
SEM analysis of various scaffolds. Low- and high-magnification of SEM images of (A, a) unmodified control scaffold without microspheres, (B, b) unmodified scaffold with microspheres, (C, c) CAP-modified scaffolds with 1 min, (D, d) 3 min and (E, e) 5 min treatment time.

**Fig 3:**
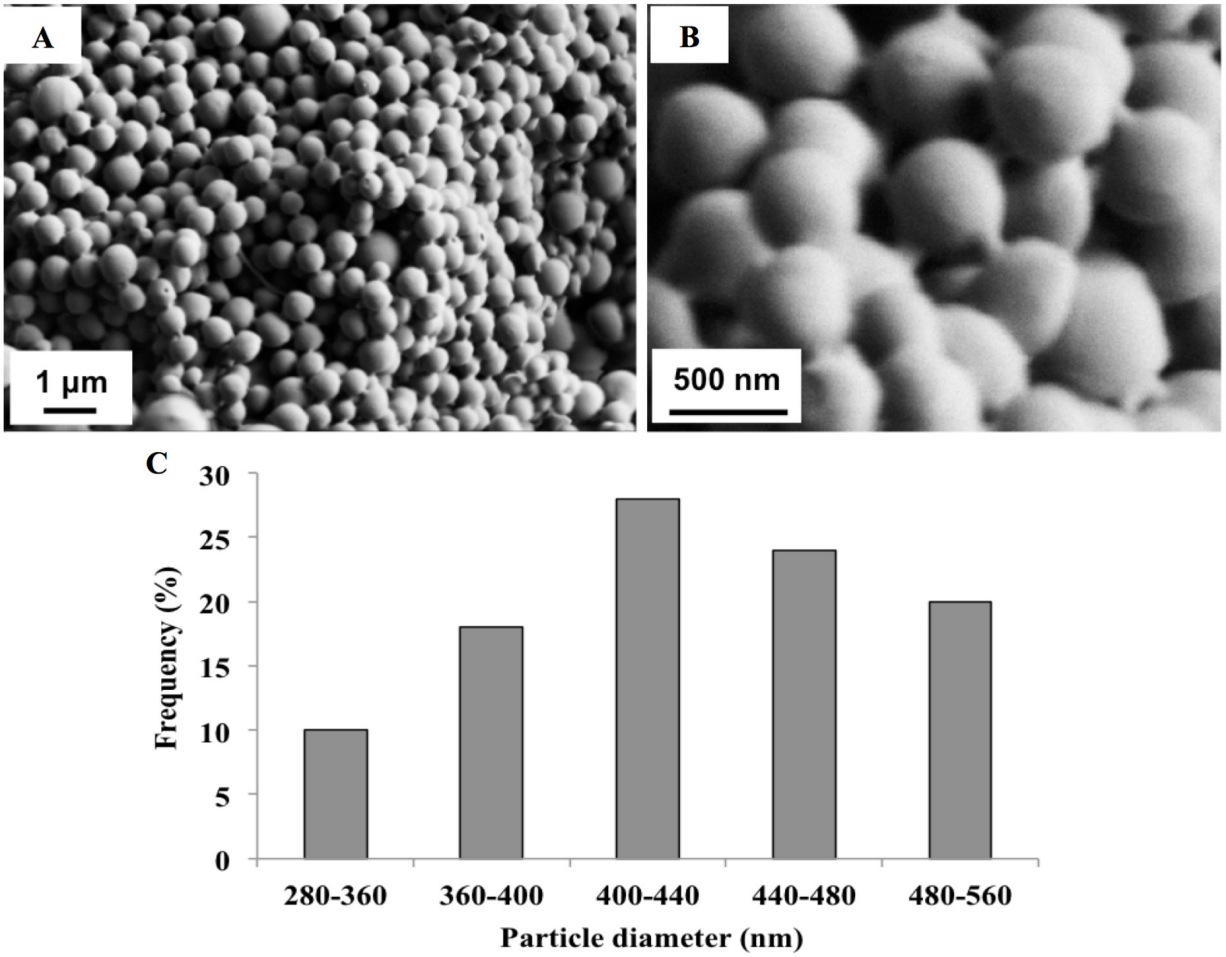
SEM images of microspheres (A-B) and their size distribution (C).

The SEM images of test samples were shown in Fig 2. Following optimized electrospinning conditions as described in our previous studies [24], electrospun fibers were successfully fabricated without beading. The fibers exhibited random orientation with uniform fiber size and interconnected pores. Homogenous microsphere distribution upon the scaffolds can be observed (Fig 2B and 2b). In addition, CAP treatment did not alter the scaffold surface morphology after 1 min (Fig 2C and 2c) and 3 min (Fig 2D and 2d) of exposure, respectively. However, 5 min CAP exposure led to scaffold degradation resulting in the fiber aggregation (Fig 2E and 2e), which indicates prolonged CAP exposure can alter scaffold surface and gross morphology.

In an effort to characterize the microsphere delivery system employed here, BSA-loaded PLGA microspheres were fabricated via a w/o/w double emulsion solvent extraction method, microscopically examined, and evaluated for bioactive factor encapsulation. Fig 3 shows the spheres have a uniform spherical morphology with a mean diameter size of 0.432 μm. In addition, the BSA-loaded microspheres exhibit a high encapsulation efficacy of 73.2% and 12.3% loading efficiency.

**Fig 4:**
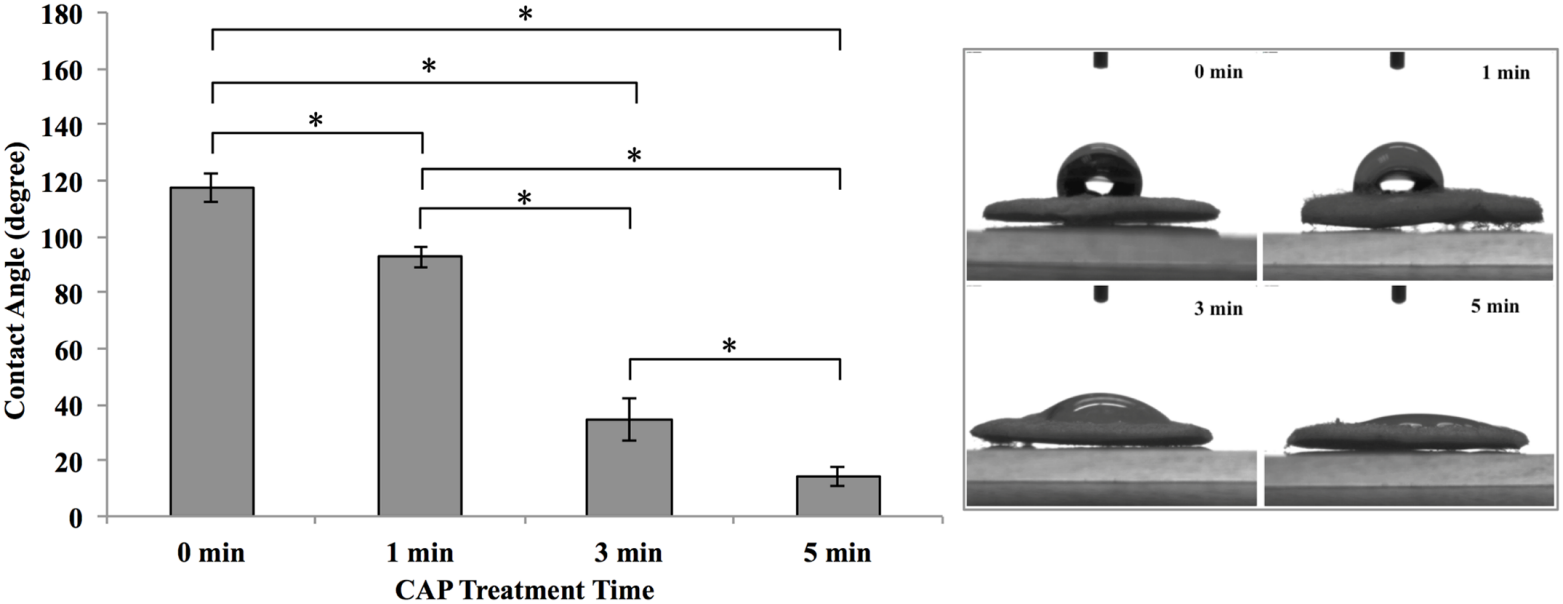
Contact angle comparison of scaffolds with and without CAP treatment. Data are mean ± standard error of the mean, n = 3; *p<0.05.

In addition to morphology, the surface properties of scaffolds including hydrophilicity and protein adsorption significantly influence scaffold performance and cytocompatibility [25]. Therefore, the hydrophilicity (contact angle) of scaffolds exposed under various CAP treatment times was measured (Fig 4). Scaffolds without CAP treatment exhibited a contact angle value of 117.3° indicating a hydrophobic surface. After CAP treatment, we found the contact angle significantly decreased and exhibited a linear dependence to exposure time. After 1 min CAP treatment, a slight decrease (92.7°) of contact angle was observed. After 3 min CAP exposure, the contact angle sharply decreased by 61.3% (34.8° contact angle) when compared to an untreated control, suggesting the induction of a hydrophilic surface. Extended exposure (up to 5 min) further increased surface hydrophilicity where the contact angle decreased to 14.2°.

**Fig 5:**
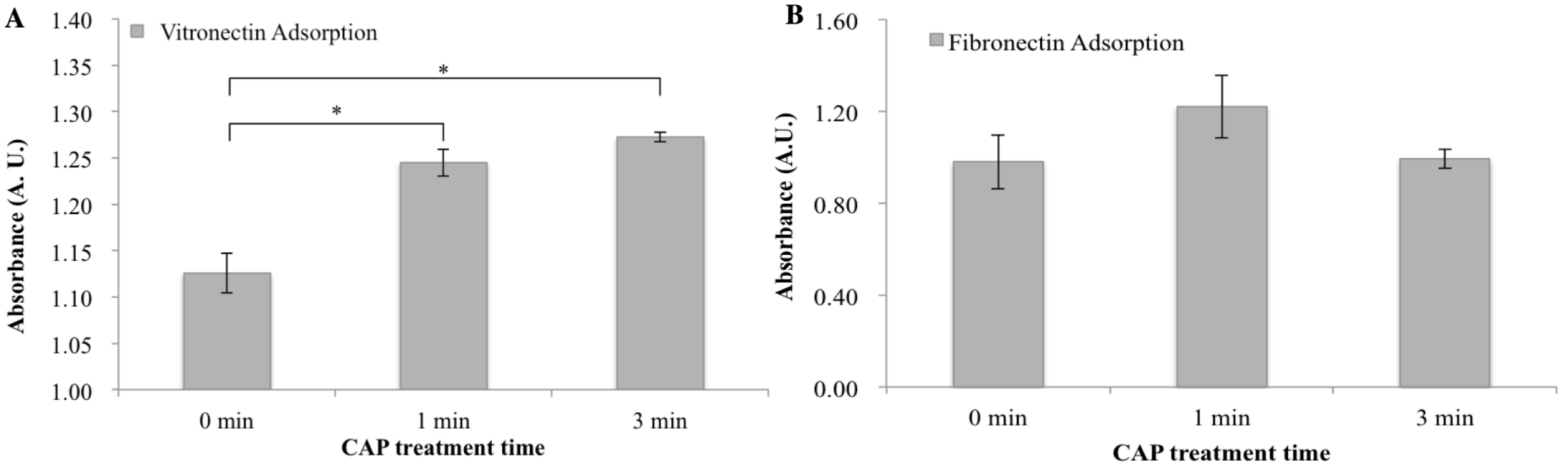
Proteins adsorption on scaffolds with different CAP treatment times. Specific (A) vitronectin and (B) fibronectin adsorption on scaffolds after 0, 1 and 3 min CAP treatment by ELISA assay, data are mean ± standard error of the mean, n = 3. *p<0.05.

Based on the contact angle results, we further investigated specific vitronectin and fibronectin adsorption on microsphere-embedded scaffolds after CAP exposure. Specifically, CAP exposure increased vitronectin adsorption by 10.6% and 13% after 1 and 3 min treatment times, respectively, when compared to an untreated control (Fig 5A). There was no significant difference in fibronectin adsorption amongst the sample groups (Fig 5B).

**Fig 6:**
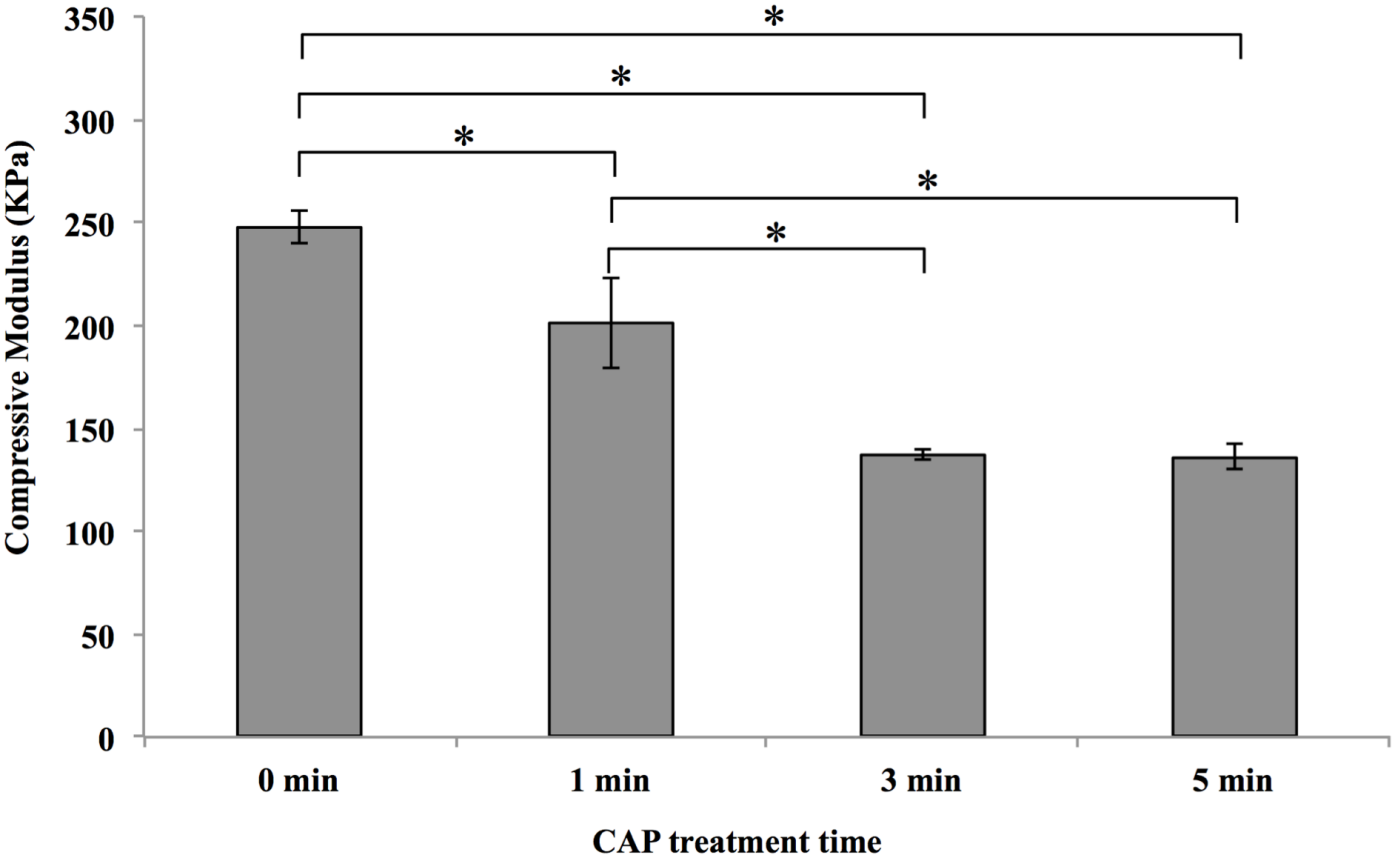
Compressive modulus of CAP modified and unmodified (0 min) scaffolds. Sample thickness is 0.5 mm, data are mean ± standard error of the mean, n = 3, *p<0.05.

Fig 6 shows the compressive modulus of CAP modified and unmodified scaffolds with embedded microspheres. The unmodified scaffold exhibited a compressive modulus of 248 KPa with CAP modification slightly decreasing the compressive modulus. Specifically, after 1 min of CAP exposure the scaffold exhibited a compressive modulus of 201 KPa. With increasing CAP treatment, the compressive modulus decreased to 137 and 136 KPa corresponding to 3 min and 5 min exposure time, respectively.

### Enhanced MSC Proliferation on Scaffolds with CAP Treatment and Microspheres

**Fig 7:**
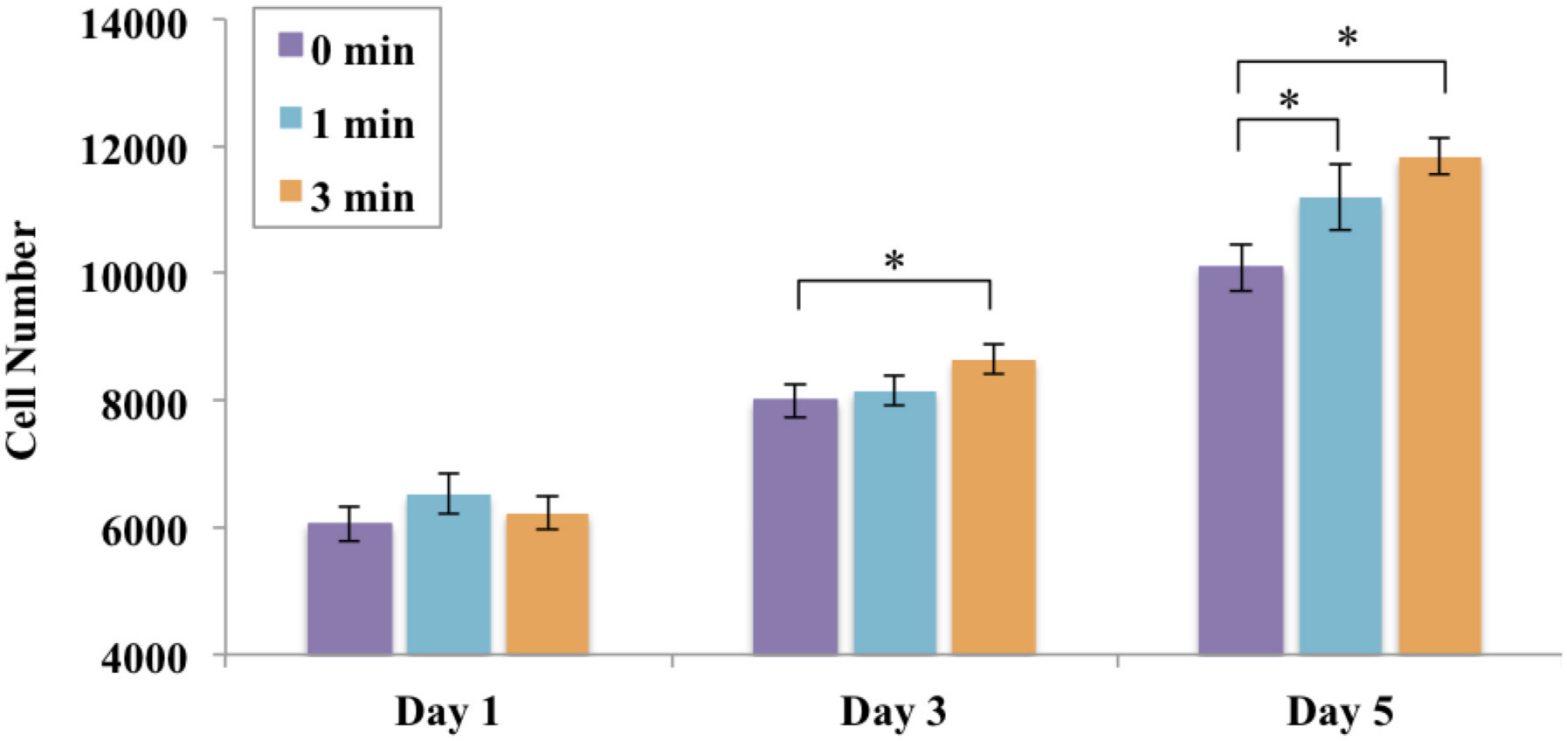
Enhanced MSC proliferation on CAP modified scaffolds after 3 and 5 days. Data are mean ± standard error of the mean, N = 3, *p<0.05.

In an effort to evaluate the effects of CAP treatment on MSC behavior, 1, 3, and 5 day cell proliferation was performed on scaffolds with embedded microspheres exposed to CAP treatment. Results show that all experimental groups exhibited excellent biocompatibility as well as increased cell proliferation over time (Fig 7). More importantly, CAP treatment further enhanced MSC proliferation, displaying a time dependent growth profile. Specifically, scaffolds exposed to CAP for 3 minutes displayed the highest cell proliferation with an increased cell number of 17.3% when compared to an untreated control (0 min) after 5 days of culture. Scaffolds exposed to 1 min CAP treatment time exhibited a 10.9% increase in cell number after 5 days.

**Fig 8:**
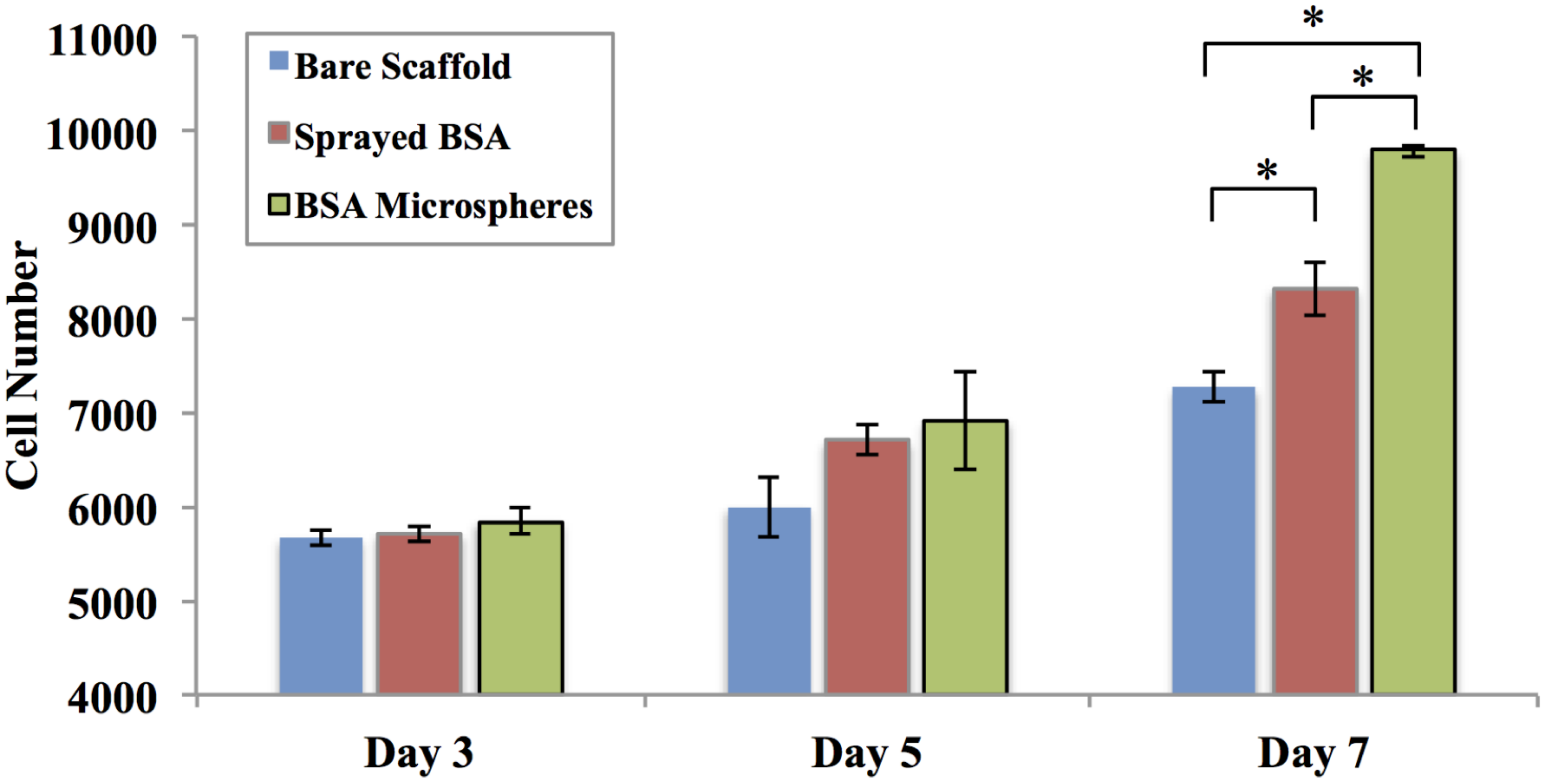
MSC proliferation after 3, 5 and 7 days culture. Samples include bare PCL scaffolds (as control), PCL scaffolds with directly sprayed BSA, and scaffolds with BSA-loaded microspheres, data are mean ± standard error of the mean, N = 3, *p<0.05.

In addition, the contribution of bioactive factor loaded microspheres on 3, 5, and 7 day MSC proliferation was also evaluated (Fig 8). Samples included electrospun fibers with BSA-loaded microspheres, directly sprayed BSA (identical amount bare BSA was directly sprayed on the surface of scaffolds without microsphere encapsulation), and bare scaffolds only (control). Here, BSA was selected as a model bioactive factor to illustrate the function of microspheres on regulating cell behavior. Results reveal that MSC number was similar amongst all groups with no significant difference after 3 and 5 days culture. When the culture time was extended to 7 days, both BSA-loaded microspheres and directly sprayed BSA groups had significantly increased cell proliferation when compared to bare scaffolds. The greatest MSC proliferation was observed on BSA-loaded microsphere scaffolds with a 14.4% and 34.5% increase when compared to BSA-sprayed and control scaffolds after 7 days of culture, respectively.

**Fig 9:**
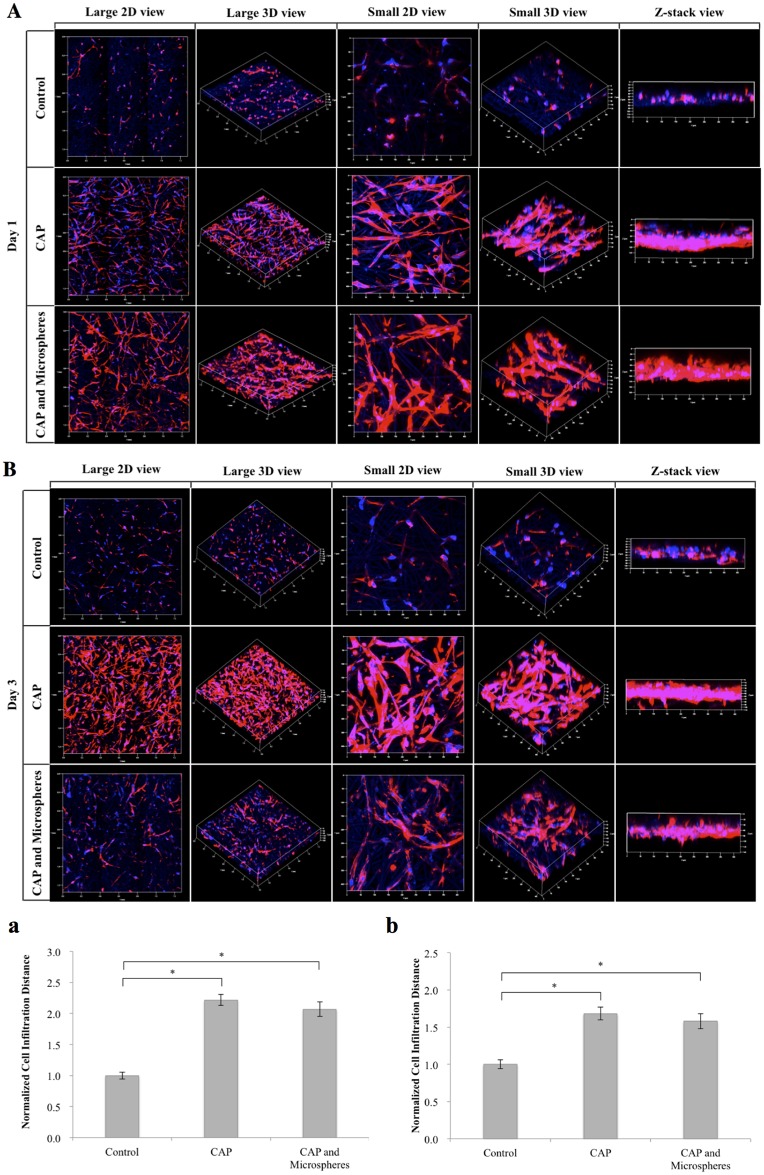
Confocal micrographs of MSCs spread and proliferate on various scaffolds. At day 1 (A) and day 3 (B) cells penetrated to scaffolds with different cells infiltration distance for 1 day (a) and 3 days (b). Improved cell proliferation and infiltration were observed on scaffolds with CAP treatment and/or microspheres incorporation compared to PCL only control group. Data are mean ± standard error of the mean, N = 3, *p<0.05.

Moreover, confocal microscopy was carried out on various MSC seeded scaffolds including bare scaffolds, scaffolds with CAP treatment, and scaffolds with both CAP treatment and BSA-loaded microspheres to evaluate 3D cell spreading and infiltration. As shown in Fig 9, excellent MSC adhesion and spreading was observed throughout the entirety of the CAP and microsphere embedded scaffolds’ surface with well-defined actin filaments (stained red by Rhodamine-Phalloidin) and nuclei (stained blue by DAPI) suggesting desirable biocompatibility of the scaffold groups. More importantly, CAP treated scaffolds (with and without microspheres) can substantially enhance 3D cell infiltration when compared with PCL scaffolds without CAP treatment after 1 and 3 days culture, respectively. Image analysis revealed a 2.22 and 2.07-fold increase of infiltration depth for 1 min and 3 min CAP treated scaffolds, respectively, when compared to untreated scaffolds at day 1. At day 3, the infiltration depth on scaffolds with 1 min and 3 min CAP treatment was 1.68 and 1.58 times relative to untreated scaffolds. There was no significant difference in cell infiltration depth amongst CAP treated groups with and without microspheres at day 1 and day 3.

### CAP and Microspheres Synergistically Improved Chondrogenic Differentiation of MSCs

**Fig 10:**
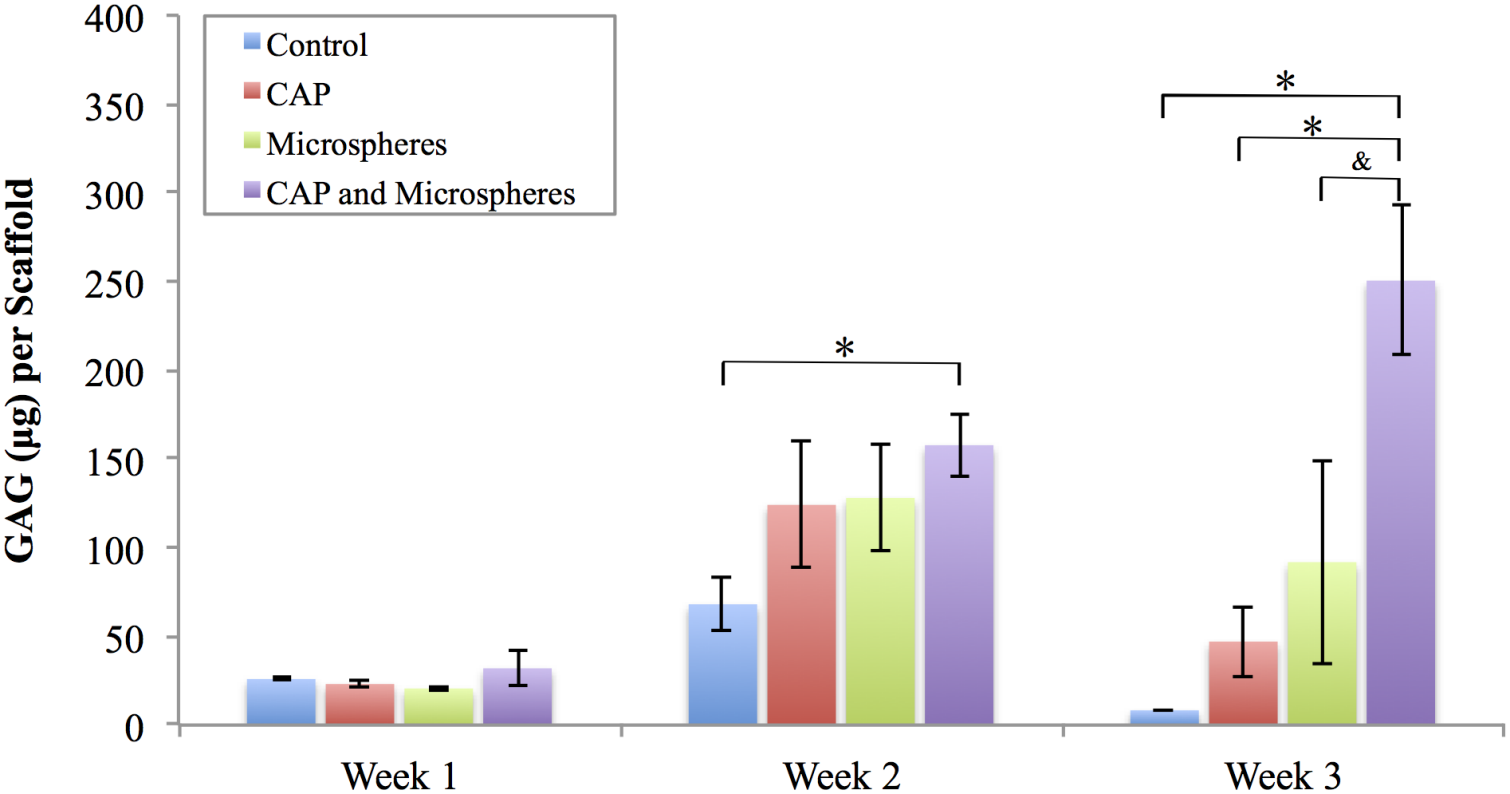
GAG secretion in various scaffolds. Enhanced GAG secretion in CAP modified microspheres incorporated scaffolds relative to controls after three weeks culture, data are mean ± standard error of the mean, N = 3, *p < 0.05, ^&^p<0.1.

GAG is a critical component of native cartilage extracellular matrix which plays a significant role in recruiting growth factors, cytokines, and water retention [26]. Enhancing GAG secretion is extremely desirable for scaffolds targeting cartilage tissue repair. Thus, we quantified deposited GAG content upon our scaffolds after culturing MSCs in chondrogenic media for 1, 2, and 3 weeks. Samples included bare PCL control, microsphere-embedded scaffolds, and CAP treated scaffolds with and without embedded microspheres. Specifically, TGF-β1 as an inducer of chondrogenesis was encapsulated within the microspheres. Results showed an increase in GAG production over time for all scaffolds in the first two weeks (Fig 10), and CAP treated groups with microspheres exhibited more GAG content than bare PCL control in week 2. After 3 weeks, scaffolds with both CAP treatment and microspheres showed the highest increase in GAG synthesis when compared to all other groups.

**Fig 11:**
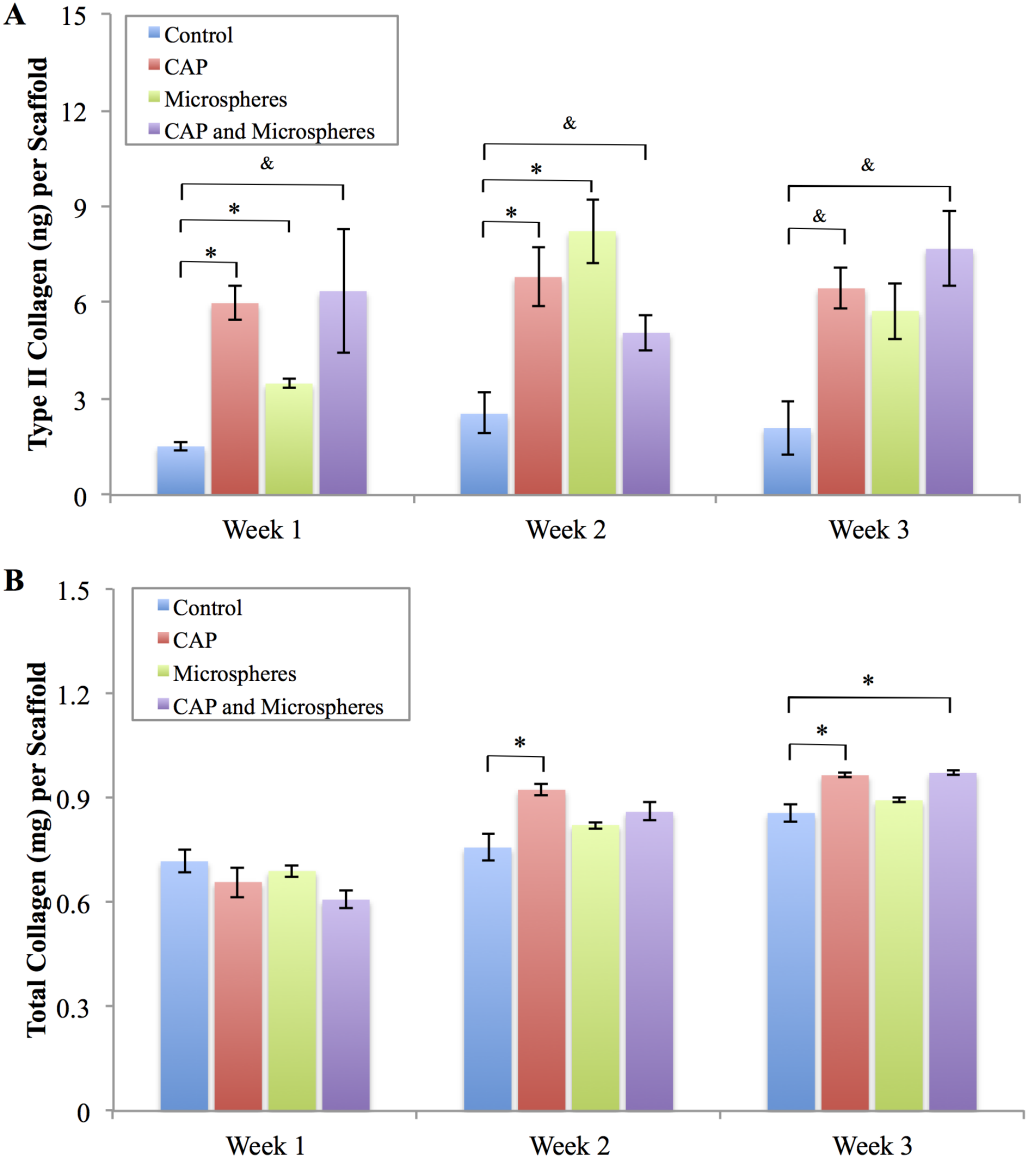
Type II collagen and total collagen synthesis. Scaffolds with CAP treated, microspheres embedded, and both CAP and microspheres modification promoted (A) type II collagen and (B) total collagen synthesis relative to bare control, data are mean ± standard error of the mean, N = 3, *p < 0.05, ^&^p<0.1.

In addition to total GAG content, type II collagen (another important indicator of MSC chondrogenesis) was evaluated. As shown in Fig 11A, type II collagen synthesis was significantly improved after each time point for the scaffolds with CAP treatment, embedded microspheres, and both CAP and embedded microsphere modification when compared to control. Additionally, total collagen quantification (Fig 11B) demonstrated that scaffolds with CAP treatment and embedded microspheres outperformed the bare control after three weeks of culture. These results help to illustrate the synergistic promotion of CAP and bioactive microspheres for regulated MSC chondrogenic differentiation *in vitro*.

## Discussion

### Integrating CAP and Electrospinning for Fabricating Bioactive Cartilage Scaffold

Electrospinning has been widely investigated in tissue engineering fields and showed promise in promoting various desired cellular activities [27–30]. However, the lack of ideal bioactive surface properties and excellent 3D cell infiltration nature within many electrospun scaffolds is a major concern typically resulting in less than ideal tissue regeneration. Even though many traditional approaches are available to modify the electrospun scaffolds as described in the introduction section, a simple, efficient, and one-step modification method is still highly desirable. Therefore, in this report, we uniquely combined CAP with electrospinning in an effort to yield a more bioactive and biomimetic scaffold for cartilage regeneration.

CAP is an emerging medical technique which holds great promise for biomedical applications, its complex composition renders it capable of remodeling and altering the scaffold microarchitecture and surface properties [22]. Our results demonstrate that CAP treatment can easily render the inherent hydrophobic surface properties of electrospun PCL scaffolds (contact angle = 134°) to more hydrophilic (contact angle = 34.8° after 3 min CAP treatment) in a one-step process as well as enhance specific protein adsorption for better cell adhesion (Figs 4 and 5). It is worth noting that CAP treatment could be precisely controlled to avoid scaffold surface damage (Fig 2). Although a slight decrease in compressive modulus after CAP treatment was observed, it still remains in the modulus range of native cartilage (0.1–2 MPa) [31]. It is postulated that the observed alteration of bulk mechanical properties is ascribed to a change in compressive strength of individual fibers. In addition, the softer surface induced by CAP treatment probably contributed partially to improved cell functions, such as proliferation and chondrogenic differentiation.

The influence of CAP on scaffold surface properties may be attributed to the introduction of oxygen-containing charged species deposited upon the scaffold surface [32]. It is well known that the CAP plume contains various reactive oxygen species. The oxygen-rich plasma treatment can introduce oxygen-containing species, such as C-O, C = O, O-C = O, C-O-O, and CO_3_, upon the polymer surface leading to reactions between the polymer chain and atomic oxygen found within the CAP plume [33]. Not only does the addition of reactive oxygen species improve material hydrophilicity, better protein adsorption leading to enhanced cell attachment and proliferation [34, 35].

Our cell proliferation results (Fig 7) confirm the promotion of cell proliferation with an increase of CAP treatment time. Other studies suggest improved cell response as a result of alterations to the surface roughness caused by CAP surface modification [22, 36]. Increases in surface roughness promote cell attachment, morphological changes, and differentiation [37]. In addition to the promotion of cell proliferation, qualitative evaluation by confocal microscopy displays excellent cell spreading and growth on CAP modified scaffolds when compared to untreated samples (Fig 9). More importantly, MSCs exhibited much deeper cellular infiltration on CAP treated scaffolds than untreated controls after 1 and 3 days of culture. Traditional electrospun scaffolds are always limited by their poor cell infiltration due to tightly compacted fibrous layers. In order to address this, many approaches have been investigated such as the incorporation of nanoparticles, enlarge microfibers, leaching embedded salt [38]. However, these conventional strategies routinely involve laborious procedures, and may influence the integrity of original scaffolds as well as impair the scaffolds’ bioactive properties. Our results indicate CAP treatment is a robust tool for the modification of electrospun scaffolds with greatly improved cell infiltration capacity. This easy one-step modification strategy may be opening a new avenue to tackle the challenge of electrospun scaffolds for various tissue engineering applications.

### Incorporating Microspheres into CAP Treated Scaffolds to Provide Sustained Bioactive Factor Environment

MSC and chondrocytes are excellent cell sources for repairing articular cartilage defects [39, 40]. When compared to chondrocytes, the use of MSCs exhibits superiority in cartilage regeneration. MSCs are readily acquired from the bone marrow or other mesenchyme-derived tissues while mature chondrocytes can only be harvested from a limited supply of healthy articular cartilage. The harvesting of MSCs does not require the use of cartilage biopsy which further lowers complications associated with donor-site morbidity [41, 42]. During the use of MSCs, some key factors are considered as potential strategies to enhance MSC-based cartilage repair. TGF-β1, a member of the TGF-β superfamily, can stimulate chondrogenesis of MSC resulting in the enhanced production of cartilaginous extracellular matrix [43]. Therefore, the development of scaffolds with controlled delivery of bioactive TGF-β1 can provide temporal and spatial control of MSC differentiation both *in vitro* and *in vivo*.

Consequently, in addition to CAP surface modification, we introduced a microsphere-based sustained bioactive factor delivery system to be used in combination with electrospun fibrous scaffolds. In order to evaluate the efficacy of bioactive microspheres, we first loaded BSA within the microspheres, and performed a 7 day cell proliferation study. Results show increased cell number at day 7 in groups containing sprayed BSA and BSA loaded microspheres when compared to control (Fig 8). In particular, the group containing BSA loaded microspheres exhibit the highest cell growth after 7 days of culture. This may be due to the rapid diffusion of bare BSA when directly spayed upon PCL fibrous scaffolds where most of the BSA would be removed during regular media exchange resulting in the absence of long-term stimulation. In contrast, microspheres incorporated with the scaffold matrix can protect BSA and allow for release of BSA for a longer term when compared to bare BSA in the scaffold.

Similarly, improved cellular response was observed when TGF-β1 was loaded within the microspheres during the 3-week differentiation study (Figs 10 and 11). A significant increase in total GAG and type II collagen was noted in the CAP treated and microsphere embedded scaffold group which can be attributed to both sustained release of TGF-β1 and CAP treatment for improved cellular function.

As a whole, our findings suggest a synergistic effect between CAP and bioactive factor encapsulated microspheres where both CAP treatment and sustained bioactive factor delivery microspheres allow for greater cell growth and elevated cellular activities. For the first time, we find CAP has the capacity in improving cell infiltrating into electrospun scaffolds and chondrogenic differentiation. Thus, the integrated techniques used in this study hold high potential in cartilage regeneration, and, not limited to cartilage application, it could be considered to create next generation of bioactive tissue-engineered scaffolds for various tissue regeneration applications.

## Conclusion

A series of novel CAP modified cartilage scaffolds were fabricated in the current study and they displayed interconnected porous topography with homogeneously distributed microspheres. Surface modification via CAP treatment in combination with bioactive factors encapsulated microspheres promoted MSC growth, 3D infiltration, and chondrogenesis. CAP treatment alone can readily enhance adhesion-mediating protein adsorption (vitronectin) on PCL scaffolds and, thus, promote MSC function. Quantitative biochemical assays confirm the enhanced synthesis of GAG, type II collagen and total collagen on microsphere embedded CAP treated scaffolds after three weeks of culture. Collectively, our work suggests this novel CAP treated scaffold can be used as a potential construct for cartilage regeneration. Furthermore, the combination of CAP technique, bioactive factor loaded microspheres and electrospinning holds great promise for a variety of tissue regeneration.

## References

[1] Adam C, Eckstein F, Milz S, Schulte E, Becker C, Putz R. The distribution of cartilage thickness in the knee-joints of old-aged individuals—measurement by A-mode ultrasound. Clinical Biomechanics. 1998;13(1):1–10. 10.1016/S0268-0033(97)85881-0 11415765

[2] Matsiko A, Levingstone TJ, O'Brien FJ. Advanced Strategies for Articular Cartilage Defect Repair. MATERIALS. 2013;6(2):637–68. 10.3390/ma6020637 28809332 PMC5452095

[3] Makris EA, Gomoll AH, Malizos KN, Hu JC, Athanasiou KA. Repair and tissue engineering techniques for articular cartilage. NATURE REVIEWS RHEUMATOLOGY. 2015;11(1):21–34. 10.1038/nrrheum.2014.157 25247412 PMC4629810

[4] Vinatier C, Mrugala D, Jorgensen C, Guicheux J, Noël D. Cartilage engineering: a crucial combination of cells, biomaterials and biofactors. Trends in Biotechnology. 2009;27(5):307–14. 10.1016/j.tibtech.2009.02.005 19329205

[5] Holmes B, Castro NJ, Zhang LG, Zussman E. Electrospun fibrous scaffolds for bone and cartilage tissue generation: recent progress and future developments. Tissue Eng Part B Rev. 2012;18(6):478–86. Epub 2012/06/29. 10.1089/ten.TEB.2012.0096 . 22738358

[6] Pham QP, Sharma U, Mikos AG. Electrospinning of polymeric nanofibers for tissue engineering applications: a review. Tissue engineering. 2006;12(5):1197–211. 10.1089/ten.2006.12.1197 16771634

[7] Li WJ, Danielson KG, Alexander PG, Tuan RS. Biological response of chondrocytes cultured in three-dimensional nanofibrous poly(epsilon-caprolactone) scaffolds. JOURNAL OF BIOMEDICAL MATERIALS RESEARCH PART A. 2003;67A(4):1105–14. 10.1002/jbm.a.10101 14624495

[8] Ciardelli G, Chiono V, Vozzi G, Pracella M, Ahluwalia A, Barbani N, et al Blends of poly-(epsilon-caprolactone) and polysaccharides in tissue engineering applications. Biomacromolecules. 2005;6(4):1961–76. 10.1021/bm0500805 16004434

[9] Ghasemi-Mobarakeh L. Surface modification of PCL nanofiber mats for tissue engineering. TISSUE ENGINEERING. 2007;13(4):916–7.

[10] Zheng R, Zhang W, Liu W, Cao Y, Zhou G, Duan H, et al The influence of Gelatin/PCL ratio and 3-D construct shape of electrospun membranes on cartilage regeneration. Biomaterials. 2014;35(1):152–64. 10.1016/j.biomaterials.2013.09.082 24135269

[11] Neves SC, Moreira Teixeira LS, Moroni L, Reis RL, Van Blitterswijk CA, Alves NM, et al Chitosan/Poly(ɛ-caprolactone) blend scaffolds for cartilage repair. Biomaterials. 2011;32(4):1068–79. 10.1016/j.biomaterials.2010.09.073 20980050

[12] Domingos M, Intranuovo F, Gloria A, Gristina R, Ambrosio L, Bártolo PJ, et al Improved osteoblast cell affinity on plasma-modified 3-D extruded PCL scaffolds. Acta biomaterialia. 2013;9(4):5997–6005. 10.1016/j.actbio.2012.12.031 23313115

[13] Chen L, Bai Y, Liao G, Peng E, Wu B, Wang Y, et al Electrospun Poly(L-lactide)/Poly(ly(osteoblast cell affinity on plasma-modd: Characterization and Biocompatibility with Human Adipose-Derived Stem Cells. PLoS ONE. 2013;8(8):e71265 10.1371/journal.pone.0071265 23990941 PMC3753307

[14] Shashurin A, Keidar M, Bronnikov S, Jurjus RA, Stepp MA. Living tissue under treatment of cold plasma atmospheric jet. Appl Phys Lett. 2008;93(18). doi: Artn 181501 10.1063/1.3020223 ISI:000260778100016.

[15] Keidar M, Shashurin A, Volotskova O, Stepp MA, Srinivasan P, Sandler A, et al Cold atmospheric plasma in cancer therapy. Physics of Plasmas. 2013;20(5):057101–8. 10.1063/1.4801516

[16] Kolb JF, Mohamed A-AH, Price RO, Swanson RJ, Bowman A, Chiavarini RL, et al Cold atmospheric pressure air plasma jet for medical applications. Applied Physics Letters. 2008;92(24):241501–3. 10.1063/1.2940325

[17] Li Y-F, Shimizu T, Zimmermann JL, Morfill GE. Cold atmospheric plasma for surface disinfection. Plasma Processes and Polymers. 2012;9(6):585–9. 10.1002/ppap.201100090

[18] Wang M, Holmes B, Cheng X, Zhu W, Keidar M, Zhang LG. Cold atmospheric plasma for selectively ablating metastatic breast cancer cells. PloS one. 2013;8(9):e73741 10.1371/journal.pone.0073741 24040051 PMC3770688

[19] Ratovitski EA, Cheng X, Yan D, Sherman JH, Canady J, Trink B, et al Anti-Cancer Therapies of 21st Century: Novel Approach to Treat Human Cancers Using Cold Atmospheric Plasma. Plasma Processes and Polymers. 2014;11(12):1128–37. 10.1002/ppap.201400071

[20] Cheng XQ, Sherman J, Murphy W, Ratovitski E, Canady J, Keidar M. The Effect of Tuning Cold Plasma Composition on Glioblastoma Cell Viability. PLOS ONE. 2014;9(5):e98652 10.1371/journal.pone.0098652 24878760 PMC4039517

[21] Stoffels E, Flikweert AJ, Stoffels WW, Kroesen GMW. Plasma needle: a non-destructive atmospheric plasma source for fine surface treatment of (bio)materials. Plasma Sources Science and Technology. 2002;11(4):383–8. 10.1088/0963-0252/11/4/304

[22] Wang M, Cheng X, Zhu W, Holmes B, Keidar M, Zhang LG. Design of biomimetic and bioactive cold plasma-modified nanostructured scaffolds for enhanced osteogenic differentiation of bone marrow-derived mesenchymal stem cells. Tissue Engineering—Part A. 2014;20(5–6):1060–71. 10.1089/ten.TEA.2013.0235 24219622

[23] Baldwin SP, Saltzman WM. Materials for protein delivery in tissue engineering. Advanced Drug Delivery Reviews. 1998;33(1–2):71–86. 10837654 10.1016/s0169-409x(98)00021-0

[24] Zhu W, Masood F, O’Brien J, Zhang LG. Highly Aligned Nanocomposite Scaffolds by Electrospinning and Electrospraying for Neural Tissue Regeneration. Nanomedicine: Nanotechnology, Biology and Medicine. 2015 10.1016/j.nano.2014.12.001 25596341

[25] Place ES, George JH, Williams CK, Stevens MM. Synthetic polymer scaffolds for tissue engineering. Chemical Society reviews. 2009;38(4):1139–51. 10.1039/b811392k 19421585

[26] Zhang L, Hu J, Athanasiou KA. The role of tissue engineering in articular cartilage repair and regeneration. Crit Rev Biomed Eng. 2009;37(1–2):1–57. Epub 2009/01/01. doi: 7a765eb33e53ff66,08b592492248b67f [pii]. 20201770 10.1615/critrevbiomedeng.v37.i1-2.10 PMC3146065

[27] Holmes B, Castro NJ, Li J, Keidar M, Zhang LG. Enhanced human bone marrow mesenchymal stem cell functions in novel 3D cartilage scaffolds with hydrogen treated multi-walled carbon nanotubes. Nanotechnology. 2013;24(36):365102-1-10. 10.1088/0957-4484/24/36/365102 23959974

[28] Yoshimoto H, Shin YM, Terai H, Vacanti JP. A biodegradable nanofiber scaffold by electrospinning and its potential for bone tissue engineering. Biomaterials. 2003;24(12):2077–82. 10.1016/S0142-9612(02)00635-X 12628828

[29] Zhu W, O'Brien C, O'Brien JR, Zhang LG. 3D nano/microfabrication techniques and nanobiomaterials for neural tissue regeneration. NANOMEDICINE. 2014;9(6):859–75. 10.2217/NNM.14.36 24981651

[30] Holmes B, Zarate A, Keidar M, Zhang LG. Enhanced Human Bone Marrow Mesenchymal Stem Cell Chondrogenic Differentiation in Electrospun Constructs with Carbon Nanomaterials. Carbon. 2016;97:1–13.

[31] Moutos FT, Freed LE, Guilak F. A biomimetic three-dimensional woven composite scaffold for functional tissue engineering of cartilage. Nature Materials. 2007;6(2):162–7. 10.1038/nmat1822 17237789

[32] Dolci LS, Focarete ML, Quiroga SD, Gherardi M, Laurita R, Liguori A, et al Carboxyl Surface Functionalization of Poly(L-lactic acid) Electrospun Nanofibers through Atmospheric Non-Thermal Plasma Affects Fibroblast Morphology. Plasma Processes and Polymers. 2014;11(3):203–13. 10.1002/ppap.201300104

[33] Chan CM, Ko TM, Hiraoka H. Polymer surface modification by plasmas and photons. Surface Science Reports. 1996;24(1–2):3–54.

[34] Kim CH, Khil MS, Kim HY, Lee HU, Jahng KY. An improved hydrophilicity via electrospinning for enhanced cell attachment and proliferation. Journal of Biomedical Materials Research—Part B Applied Biomaterials. 2006;78(2):283–90. 10.1002/jbm.b.30484 16362963

[35] Moffa M, Polini A, Sciancalepore AG, Persano L, Mele E, Passione LG, et al Microvascular endothelial cell spreading and proliferation on nanofibrous scaffolds by polymer blends with enhanced wettability. Soft Matter. 2013;9(23):5529–39. 10.1039/c3sm50328c

[36] Desmet T, Morent R, De Geyter N, Leys C, Schacht E, Dubruel P. Nonthermal plasma technology as a versatile strategy for polymeric biomaterials surface modification: a review. Biomacromolecules. 2009;10(9):2351–78. 10.1021/bm900186s 19655722

[37] Moroni L, de Wijn JR, van Blitterswijk CA. Integrating novel technologies to fabricate smart scaffolds. Journal of Biomaterials Science, Polymer Edition. 2008;19(5):543- 10.1163/156856208784089571 18419938

[38] Blakeney BA, Tambralli A, Anderson JM, Andukuri A, Lim D-J, Dean DR, et al Cell infiltration and growth in a low density, uncompressed three-dimensional electrospun nanofibrous scaffold. Biomaterials. 2011;32(6):1583–90. 10.1016/j.biomaterials.2010.10.056 21112625 PMC3023580

[39] Mats B, Lars P, Eva S, Gren J, Tommi T, Anders L. Articular Cartilage Engineering with Autologous Chondrocyte Transplantation A Review of Recent Developments. The Journal of Bone & Joint Surgery. 2003;85(suppl_3):109–15. 10.2106/00004623-200300003-00017 12925617

[40] Toh WS, Foldager CB, Pei M, Hui JHP. Advances in Mesenchymal Stem Cell-based Strategies for Cartilage Repair and Regeneration. Stem Cell Reviews and Reports. 2014 10.1007/s12015-014-9526-z 24869958

[41] Madry H, Rey-Rico A, Venkatesan JK, Johnstone B, Cucchiarini M. Transforming Growth Factor Beta-Releasing Scaffolds for Cartilage Tissue Engineering. Tissue Engineering, Part B: Reviews. 2014;20(2):106–25. 10.1089/ten.teb.2013.0271 23815376

[42] Nöth U, Steinert AF, Tuan RS. Technology insight: adult mesenchymal stem cells for osteoarthritis therapy. Nature clinical practice Rheumatology. 2008;4(7):371–80. 10.1038/ncprheum0816 18477997

[43] Li WJ, Tuli R, Okafor C, Derfoul A, Danielson KG, Hall DJ, et al A three-dimensional nanofibrous scaffold for cartilage tissue engineering using human mesenchymal stem cells. Biomaterials. 2005;26(6):599–609. 10.1016/j.biomaterials.2004.03.005 15282138

